# [5,10,15,20-Tetra­kis(4-meth­oxy­phen­yl)porphyrinato-κ^4^ *N*,*N*′,*N*′′,*N*′′′](trifluoro­methane­sulfonato-κ*O*)­iron(III)

**DOI:** 10.1107/S1600536811001395

**Published:** 2011-01-29

**Authors:** Nan Xu, Douglas R. Powell, George B. Richter-Addo

**Affiliations:** a Department of Chemistry and Biochemistry, University of Oklahoma, 101 Stephenson Parkway, Norman, OK 73019-5251, USA

## Abstract

The title compound, [Fe(CF_3_O_3_S)(C_48_H_36_N_4_O_4_)], is a five-coordinate iron(III) porphyrin complex with a trifluoro­methane­sulfonate anion as an axial ligand. The Fe^III^ atom is displaced by 0.40 (1) Å towards the trifluoro­methane­sulfonate anion from the 24-atom mean plane of the porphyrin. The average Fe—N_p_ distance is 2.044 (2) Å and the Fe—O distance is 2.001 (2) Å.

## Related literature

For the structures of related porphyrin (‘picket-fence’, tetra­phenyl­porphyrin, octa­ethyl­porphyrin) derivatives, see: González & Wilson (1994 ▶); Gismelseed *et al.* (1990 ▶); Xu *et al.* (2008 ▶).
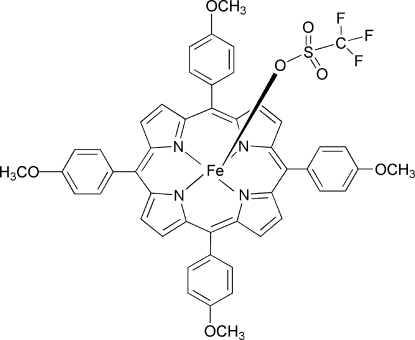


## Experimental

### 

#### Crystal data

[Fe(CF_3_O_3_S)(C_48_H_36_N_4_O_4_)]
*M* *_r_* = 937.73
Triclinic, 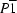

*a* = 12.5265 (14) Å
*b* = 13.2725 (16) Å
*c* = 14.0220 (17) Å
α = 90.080 (2)°
β = 112.534 (2)°
γ = 103.483 (3)°
*V* = 2083.2 (4) Å^3^
*Z* = 2
Mo *K*α radiation
μ = 0.49 mm^−1^
*T* = 100 K
0.49 × 0.38 × 0.19 mm

#### Data collection

Bruker SMART APEX CCD diffractometer
Absorption correction: multi-scan (*SADABS*; Sheldrick, 2001 ▶) *T*_min_ = 0.797, *T*_max_ = 0.913
25055 measured reflections
8187 independent reflections
6693 reflections with *I* > 2σ(*I*)
*R*_int_ = 0.048

#### Refinement

*R*[*F* ^2^ > 2σ(*F* ^2^)] = 0.055
*wR*(*F* ^2^) = 0.155
*S* = 1.00
8187 reflections
586 parameters
H-atom parameters constrained
Δρ_max_ = 1.62 e Å^−3^
Δρ_min_ = −0.83 e Å^−3^

### 

Data collection: *SMART* (Bruker, 2007 ▶); cell refinement: *SAINT* (Bruker, 2007 ▶); data reduction: *SAINT*; program(s) used to solve structure: *SHELXTL* (Sheldrick, 2008 ▶); program(s) used to refine structure: *SHELXTL*; molecular graphics: *SHELXTL*; software used to prepare material for publication: *SHELXTL*.

## Supplementary Material

Crystal structure: contains datablocks I, global. DOI: 10.1107/S1600536811001395/bv2171sup1.cif

Structure factors: contains datablocks I. DOI: 10.1107/S1600536811001395/bv2171Isup2.hkl

Additional supplementary materials:  crystallographic information; 3D view; checkCIF report

## Figures and Tables

**Fig. 1.**
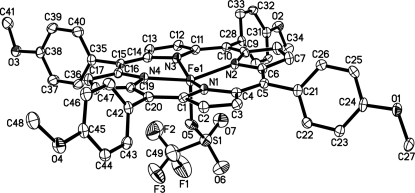
The molecular structure of (T(p-OMe)PP)Fe(OSO2CF3). Displacement ellipsoids are drawn at the 50% probability level. H atoms are omitted for clarity.

## supplementary crystallographic information

### Comment

In this paper, we report the structure of the five-coordinate compound (5,10,15,20-tetrakis(4-methoxyphenyl)porphyrinato) (trifluoromethanesulfonato)iron(III). Other trifluoromethanesulfonato iron porphyrin derivatives have been reported previously: The (T_piv_PP)Fe(OSO_2_CF_3_)(H_2_O) compound is six-coordinate at Fe, and the (TPP)Fe(OSO_2_CF_3_) and (OEP)Fe(OSO_2_CF_3_) compounds are five-coordinate at Fe (González *et al.* 1994, Gismelseed *et al.* 1990, and Xu *et al.* 2008).

The molecular structure of (5,10,15,20-tetrakis(4-methoxyphenyl)porphyrinato)(trifluoromethanesulfonato)iron(III) is shown in Fig. 1. The porphyrin core of the compound is slightly saddle shaped. The iron atom is displaced by 0.40 (1) Å from the 24-atom mean porphyrin plane toward the trifluoromethanesulfonate anion. The trifluoromethanesulfonate anion binds to the iron center through one of its sulfonato oxygen atoms. The average Fe—N_p_ distance is 2.044 (2) Å and the Fe—O distance is 2.001 (2) Å. The bond angle of the Fe—O—S linkage is 137.11 (13)°.

### Experimental

To a toluene solution (20 ml) of (T(*p*-OMe)PP)FeCl (0.025 g, 0.030 mmol) was added silver trifluoromethanesulfonate (0.009 g, 0.033 mmol) (purchased from Aldrich Chemical Company and used as received) under N_2_. After stirring for 2 h, the resulting mixture was filtered and dried under vacuum. A suitable purple prism-shaped crystal was grown by slow evaporation of a dichloromethane-hexane (1:1) solution of the complex at room temperature under N_2_.

### Refinement

The hydrogen atoms were placed in calculated positions with C—H = 0.95 Å for aromatic carbons, 0.98 Å for methyl carbons and were refined using a riding model with *U*_iso_ = 1.2 *U*_eq_(C) for phenyl H atoms, *U*_iso_ = 1.5 *U*_eq_(C) for methyl H atoms.

### Crystal data

**Table:**

[Fe(CF_3_O_3_S)(C_48_H_36_N_4_O_4_)]	*Z* = 2
*M**_r_* = 937.73	*F*(000) = 966
Triclinic, *P*1	*D*_x_ = 1.495 Mg m^−^^3^
Hall symbol: -P 1	Mo *K*α radiation, λ = 0.71073 Å
*a* = 12.5265 (14) Å	Cell parameters from 5786 reflections
*b* = 13.2725 (16) Å	θ = 2.4–28.2°
*c* = 14.0220 (17) Å	µ = 0.49 mm^−^^1^
α = 90.080 (2)°	*T* = 100 K
β = 112.534 (2)°	Prism, purple
γ = 103.483 (3)°	0.49 × 0.38 × 0.19 mm
*V* = 2083.2 (4) Å^3^	

### Data collection

**Table:**

Bruker SMART APEX CCD diffractometer	8187 independent reflections
Radiation source: fine-focus sealed tube	6693 reflections with *I* > 2σ(*I*)
graphite	*R*_int_ = 0.048
φ and ω scans	θ_max_ = 26.0°, θ_min_ = 1.6°
Absorption correction: multi-scan (*SADABS*; Sheldrick, 2001)	*h* = −15→15
*T*_min_ = 0.797, *T*_max_ = 0.913	*k* = −16→16
25055 measured reflections	*l* = −17→17

### Refinement

**Table:**

Refinement on *F*^2^	Primary atom site location: structure-invariant direct methods
Least-squares matrix: full	Secondary atom site location: difference Fourier map
*R*[*F*^2^ > 2σ(*F*^2^)] = 0.055	Hydrogen site location: inferred from neighbouring sites
*wR*(*F*^2^) = 0.155	H-atom parameters constrained
*S* = 1.00	*w* = 1/[σ^2^(*F*_o_^2^) + (0.082*P*)^2^ + 3.420*P*] where *P* = (*F*_o_^2^ + 2*F*_c_^2^)/3
8187 reflections	(Δ/σ)_max_ < 0.001
586 parameters	Δρ_max_ = 1.62 e Å^−^^3^
0 restraints	Δρ_min_ = −0.83 e Å^−^^3^

### Fractional atomic coordinates and isotropic or equivalent isotropic displacement parameters (Å^2^)

**Table:**

	*x*	*y*	*z*	*U*_iso_*/*U*_eq_	
Fe1	0.42277 (4)	0.30132 (3)	0.34621 (3)	0.01581 (13)	
S1	0.59702 (8)	0.21512 (7)	0.26618 (7)	0.0276 (2)	
F1	0.6429 (3)	0.2189 (3)	0.1011 (2)	0.0762 (10)	
F2	0.4999 (3)	0.2899 (3)	0.0886 (2)	0.0750 (9)	
F3	0.4704 (2)	0.1228 (2)	0.0792 (2)	0.0661 (8)	
O1	0.7771 (2)	0.05917 (18)	0.96773 (17)	0.0244 (5)	
O2	1.09252 (19)	0.81551 (17)	0.47718 (18)	0.0250 (5)	
O3	0.0686 (2)	0.55562 (17)	−0.26646 (17)	0.0251 (5)	
O4	−0.27784 (19)	−0.19225 (17)	0.18668 (18)	0.0257 (5)	
O5	0.4856 (2)	0.21102 (17)	0.27749 (17)	0.0240 (5)	
O6	0.6331 (3)	0.1210 (2)	0.2908 (2)	0.0445 (7)	
O7	0.6838 (2)	0.3117 (2)	0.3069 (2)	0.0397 (7)	
N1	0.3634 (2)	0.19301 (19)	0.42998 (18)	0.0146 (5)	
N2	0.5656 (2)	0.36534 (19)	0.48099 (18)	0.0154 (5)	
N3	0.4583 (2)	0.43788 (19)	0.28462 (19)	0.0166 (5)	
N4	0.2496 (2)	0.27372 (19)	0.24044 (19)	0.0152 (5)	
C1	0.2525 (2)	0.1225 (2)	0.3977 (2)	0.0157 (6)	
C2	0.2513 (3)	0.0513 (2)	0.4738 (2)	0.0182 (6)	
H2	0.1857	−0.0039	0.4703	0.022*	
C3	0.3616 (3)	0.0774 (2)	0.5521 (2)	0.0177 (6)	
H3	0.3878	0.0429	0.6130	0.021*	
C4	0.4310 (3)	0.1665 (2)	0.5259 (2)	0.0157 (6)	
C5	0.5448 (2)	0.2237 (2)	0.5926 (2)	0.0150 (6)	
C6	0.6047 (3)	0.3192 (2)	0.5722 (2)	0.0165 (6)	
C7	0.7186 (3)	0.3820 (2)	0.6418 (2)	0.0216 (7)	
H7	0.7639	0.3685	0.7101	0.026*	
C8	0.7494 (3)	0.4639 (2)	0.5924 (2)	0.0225 (7)	
H8	0.8206	0.5184	0.6196	0.027*	
C9	0.6556 (3)	0.4539 (2)	0.4916 (2)	0.0181 (6)	
C10	0.6606 (3)	0.5193 (2)	0.4154 (2)	0.0167 (6)	
C11	0.5679 (3)	0.5114 (2)	0.3185 (2)	0.0168 (6)	
C12	0.5725 (3)	0.5767 (2)	0.2385 (2)	0.0198 (6)	
H12	0.6381	0.6319	0.2418	0.024*	
C13	0.4656 (3)	0.5449 (2)	0.1569 (2)	0.0205 (7)	
H13	0.4422	0.5741	0.0925	0.025*	
C14	0.3944 (3)	0.4595 (2)	0.1855 (2)	0.0177 (6)	
C15	0.2784 (3)	0.4063 (2)	0.1219 (2)	0.0170 (6)	
C16	0.2111 (3)	0.3202 (2)	0.1490 (2)	0.0171 (6)	
C17	0.0911 (3)	0.2663 (2)	0.0841 (2)	0.0197 (6)	
H17	0.0449	0.2821	0.0171	0.024*	
C18	0.0557 (3)	0.1886 (2)	0.1362 (2)	0.0193 (6)	
H18	−0.0204	0.1403	0.1129	0.023*	
C19	0.1546 (3)	0.1926 (2)	0.2334 (2)	0.0160 (6)	
C20	0.1537 (3)	0.1212 (2)	0.3061 (2)	0.0162 (6)	
C21	0.6062 (2)	0.1789 (2)	0.6907 (2)	0.0166 (6)	
C22	0.6269 (3)	0.0815 (2)	0.6880 (2)	0.0175 (6)	
H22	0.6016	0.0429	0.6227	0.021*	
C23	0.6839 (3)	0.0380 (2)	0.7783 (2)	0.0187 (6)	
H23	0.6977	−0.0290	0.7745	0.022*	
C24	0.7202 (3)	0.0945 (2)	0.8742 (2)	0.0195 (6)	
C25	0.6996 (3)	0.1914 (2)	0.8791 (2)	0.0197 (6)	
H25	0.7243	0.2295	0.9446	0.024*	
C26	0.6427 (3)	0.2337 (2)	0.7888 (2)	0.0183 (6)	
H26	0.6282	0.3004	0.7930	0.022*	
C27	0.8137 (3)	−0.0341 (3)	0.9644 (3)	0.0271 (7)	
H27A	0.7433	−0.0918	0.9287	0.041*	
H27B	0.8565	−0.0494	1.0353	0.041*	
H27C	0.8666	−0.0252	0.9269	0.041*	
C28	0.7758 (3)	0.5982 (2)	0.4346 (2)	0.0161 (6)	
C29	0.8714 (3)	0.5651 (2)	0.4319 (2)	0.0202 (7)	
H29	0.8638	0.4926	0.4216	0.024*	
C30	0.9782 (3)	0.6349 (2)	0.4436 (2)	0.0186 (6)	
H30	1.0421	0.6105	0.4396	0.022*	
C31	0.9904 (3)	0.7403 (2)	0.4613 (2)	0.0189 (6)	
C32	0.8951 (3)	0.7751 (2)	0.4638 (2)	0.0195 (6)	
H32	0.9031	0.8475	0.4750	0.023*	
C33	0.7883 (3)	0.7046 (2)	0.4499 (2)	0.0201 (6)	
H33	0.7232	0.7290	0.4509	0.024*	
C34	1.1835 (3)	0.7814 (3)	0.4578 (3)	0.0332 (8)	
H34A	1.2115	0.7313	0.5066	0.050*	
H34B	1.2505	0.8415	0.4671	0.050*	
H34C	1.1507	0.7483	0.3866	0.050*	
C35	0.2212 (3)	0.4458 (2)	0.0198 (2)	0.0177 (6)	
C36	0.2233 (3)	0.4045 (3)	−0.0703 (3)	0.0267 (7)	
H36	0.2605	0.3491	−0.0673	0.032*	
C37	0.1721 (3)	0.4426 (3)	−0.1642 (3)	0.0287 (8)	
H37	0.1745	0.4134	−0.2250	0.034*	
C38	0.1172 (3)	0.5235 (2)	−0.1702 (2)	0.0212 (7)	
C39	0.1138 (3)	0.5647 (3)	−0.0813 (3)	0.0290 (8)	
H39	0.0766	0.6200	−0.0843	0.035*	
C40	0.1649 (3)	0.5252 (3)	0.0127 (3)	0.0278 (8)	
H40	0.1610	0.5533	0.0733	0.033*	
C41	0.0405 (3)	0.6529 (3)	−0.2709 (3)	0.0325 (8)	
H41A	−0.0270	0.6479	−0.2504	0.049*	
H41B	0.0189	0.6728	−0.3418	0.049*	
H41C	0.1100	0.7057	−0.2235	0.049*	
C42	0.0408 (3)	0.0381 (2)	0.2828 (2)	0.0174 (6)	
C43	0.0337 (3)	−0.0649 (2)	0.2547 (2)	0.0204 (7)	
H43	0.1031	−0.0833	0.2560	0.024*	
C44	−0.0729 (3)	−0.1404 (2)	0.2252 (2)	0.0211 (7)	
H44	−0.0765	−0.2102	0.2062	0.025*	
C45	−0.1751 (3)	−0.1142 (2)	0.2231 (2)	0.0193 (6)	
C46	−0.1682 (3)	−0.0132 (3)	0.2556 (2)	0.0221 (7)	
H46	−0.2364	0.0047	0.2579	0.026*	
C47	−0.0604 (3)	0.0612 (2)	0.2847 (2)	0.0213 (7)	
H47	−0.0559	0.1303	0.3067	0.026*	
C48	−0.3848 (3)	−0.1651 (3)	0.1751 (3)	0.0298 (8)	
H48A	−0.3828	−0.1472	0.2437	0.045*	
H48B	−0.4533	−0.2242	0.1391	0.045*	
H48C	−0.3922	−0.1051	0.1346	0.045*	
C49	0.5495 (4)	0.2124 (4)	0.1254 (3)	0.0484 (11)	

### Atomic displacement parameters (Å^2^)

**Table:**

	*U*^11^	*U*^22^	*U*^33^	*U*^12^	*U*^13^	*U*^23^
Fe1	0.0136 (2)	0.0161 (2)	0.0146 (2)	0.00055 (16)	0.00399 (17)	0.00446 (16)
S1	0.0271 (4)	0.0292 (5)	0.0285 (5)	0.0072 (4)	0.0128 (4)	0.0045 (4)
F1	0.0607 (17)	0.123 (3)	0.0529 (17)	−0.0032 (17)	0.0468 (15)	−0.0094 (17)
F2	0.102 (2)	0.091 (2)	0.0490 (17)	0.052 (2)	0.0317 (17)	0.0329 (16)
F3	0.0576 (16)	0.089 (2)	0.0392 (15)	−0.0046 (15)	0.0190 (13)	−0.0201 (14)
O1	0.0278 (12)	0.0260 (12)	0.0162 (11)	0.0093 (10)	0.0036 (9)	0.0065 (9)
O2	0.0169 (11)	0.0203 (12)	0.0372 (14)	0.0004 (9)	0.0125 (10)	0.0049 (10)
O3	0.0341 (13)	0.0261 (12)	0.0159 (11)	0.0140 (10)	0.0071 (10)	0.0088 (9)
O4	0.0186 (11)	0.0220 (12)	0.0294 (13)	−0.0055 (9)	0.0081 (10)	0.0017 (10)
O5	0.0270 (12)	0.0186 (11)	0.0254 (12)	−0.0027 (9)	0.0144 (10)	−0.0034 (9)
O6	0.0469 (16)	0.0382 (16)	0.0567 (19)	0.0203 (13)	0.0239 (15)	0.0111 (14)
O7	0.0303 (13)	0.0364 (15)	0.0507 (17)	0.0001 (11)	0.0192 (13)	−0.0005 (13)
N1	0.0128 (11)	0.0153 (12)	0.0133 (12)	0.0018 (9)	0.0034 (10)	0.0032 (10)
N2	0.0127 (11)	0.0166 (12)	0.0148 (12)	0.0022 (10)	0.0043 (10)	0.0044 (10)
N3	0.0151 (12)	0.0152 (12)	0.0175 (13)	0.0028 (10)	0.0050 (10)	0.0056 (10)
N4	0.0138 (11)	0.0145 (12)	0.0157 (12)	0.0020 (10)	0.0049 (10)	0.0042 (10)
C1	0.0138 (13)	0.0144 (14)	0.0194 (15)	0.0027 (11)	0.0075 (12)	0.0024 (12)
C2	0.0170 (14)	0.0154 (15)	0.0188 (15)	−0.0001 (12)	0.0058 (12)	0.0031 (12)
C3	0.0207 (15)	0.0158 (15)	0.0169 (15)	0.0038 (12)	0.0082 (12)	0.0055 (12)
C4	0.0170 (14)	0.0152 (14)	0.0163 (15)	0.0039 (12)	0.0079 (12)	0.0023 (11)
C5	0.0145 (13)	0.0177 (15)	0.0143 (14)	0.0048 (11)	0.0067 (11)	0.0033 (11)
C6	0.0169 (14)	0.0162 (15)	0.0152 (15)	0.0024 (12)	0.0061 (12)	0.0032 (11)
C7	0.0177 (15)	0.0251 (17)	0.0170 (15)	0.0005 (13)	0.0044 (12)	0.0036 (13)
C8	0.0190 (15)	0.0227 (16)	0.0201 (16)	−0.0015 (13)	0.0057 (13)	0.0035 (13)
C9	0.0154 (14)	0.0205 (15)	0.0162 (15)	−0.0001 (12)	0.0066 (12)	−0.0020 (12)
C10	0.0165 (14)	0.0118 (14)	0.0191 (15)	0.0003 (11)	0.0061 (12)	0.0013 (11)
C11	0.0159 (14)	0.0145 (14)	0.0199 (15)	0.0029 (11)	0.0073 (12)	0.0033 (12)
C12	0.0186 (15)	0.0156 (15)	0.0244 (17)	0.0028 (12)	0.0084 (13)	0.0069 (12)
C13	0.0222 (15)	0.0200 (16)	0.0197 (16)	0.0059 (13)	0.0083 (13)	0.0084 (12)
C14	0.0199 (14)	0.0152 (14)	0.0184 (15)	0.0046 (12)	0.0080 (12)	0.0049 (12)
C15	0.0192 (14)	0.0166 (15)	0.0150 (15)	0.0060 (12)	0.0058 (12)	0.0042 (12)
C16	0.0170 (14)	0.0183 (15)	0.0155 (15)	0.0061 (12)	0.0051 (12)	0.0037 (12)
C17	0.0166 (14)	0.0224 (16)	0.0165 (15)	0.0042 (12)	0.0029 (12)	0.0044 (12)
C18	0.0135 (13)	0.0218 (16)	0.0184 (16)	0.0025 (12)	0.0028 (12)	0.0024 (12)
C19	0.0149 (14)	0.0149 (14)	0.0169 (15)	0.0032 (11)	0.0052 (12)	0.0015 (11)
C20	0.0142 (13)	0.0144 (14)	0.0191 (15)	0.0011 (11)	0.0070 (12)	0.0010 (12)
C21	0.0112 (13)	0.0206 (15)	0.0165 (15)	0.0007 (11)	0.0059 (11)	0.0055 (12)
C22	0.0152 (14)	0.0179 (15)	0.0177 (15)	0.0015 (12)	0.0060 (12)	0.0003 (12)
C23	0.0163 (14)	0.0180 (15)	0.0197 (16)	0.0033 (12)	0.0051 (12)	0.0042 (12)
C24	0.0148 (14)	0.0275 (17)	0.0151 (15)	0.0056 (12)	0.0045 (12)	0.0097 (13)
C25	0.0213 (15)	0.0218 (16)	0.0132 (15)	0.0005 (12)	0.0067 (12)	−0.0009 (12)
C26	0.0175 (14)	0.0179 (15)	0.0199 (16)	0.0041 (12)	0.0081 (12)	0.0022 (12)
C27	0.0313 (18)	0.0237 (17)	0.0212 (17)	0.0118 (14)	0.0022 (14)	0.0078 (14)
C28	0.0160 (14)	0.0170 (15)	0.0127 (14)	0.0004 (12)	0.0049 (11)	0.0036 (11)
C29	0.0216 (15)	0.0157 (15)	0.0211 (16)	0.0026 (12)	0.0074 (13)	0.0031 (12)
C30	0.0169 (14)	0.0170 (15)	0.0240 (16)	0.0075 (12)	0.0085 (12)	0.0053 (12)
C31	0.0168 (14)	0.0190 (15)	0.0183 (15)	0.0001 (12)	0.0068 (12)	0.0059 (12)
C32	0.0214 (15)	0.0131 (14)	0.0228 (16)	0.0029 (12)	0.0083 (13)	0.0009 (12)
C33	0.0203 (15)	0.0199 (16)	0.0214 (16)	0.0048 (12)	0.0098 (13)	0.0022 (12)
C34	0.0259 (17)	0.0298 (19)	0.048 (2)	0.0055 (15)	0.0195 (17)	0.0093 (16)
C35	0.0160 (14)	0.0154 (14)	0.0179 (15)	0.0001 (11)	0.0048 (12)	0.0038 (12)
C36	0.0357 (19)	0.0292 (18)	0.0211 (17)	0.0199 (15)	0.0108 (14)	0.0073 (14)
C37	0.041 (2)	0.0329 (19)	0.0177 (17)	0.0203 (16)	0.0114 (15)	0.0047 (14)
C38	0.0235 (15)	0.0219 (16)	0.0162 (15)	0.0056 (13)	0.0057 (13)	0.0072 (12)
C39	0.0392 (19)	0.0282 (18)	0.0219 (17)	0.0206 (16)	0.0074 (15)	0.0066 (14)
C40	0.041 (2)	0.0302 (18)	0.0161 (16)	0.0180 (16)	0.0099 (15)	0.0048 (14)
C41	0.044 (2)	0.0240 (18)	0.0218 (18)	0.0121 (16)	0.0031 (16)	0.0075 (14)
C42	0.0145 (13)	0.0190 (15)	0.0138 (14)	−0.0007 (12)	0.0031 (11)	0.0036 (12)
C43	0.0189 (15)	0.0219 (16)	0.0194 (16)	0.0050 (12)	0.0066 (12)	0.0065 (12)
C44	0.0216 (15)	0.0150 (15)	0.0212 (16)	0.0019 (12)	0.0043 (13)	0.0038 (12)
C45	0.0160 (14)	0.0200 (15)	0.0163 (15)	−0.0025 (12)	0.0045 (12)	0.0060 (12)
C46	0.0162 (14)	0.0267 (17)	0.0230 (16)	0.0027 (13)	0.0090 (13)	0.0028 (13)
C47	0.0206 (15)	0.0184 (15)	0.0234 (17)	0.0023 (12)	0.0087 (13)	0.0019 (13)
C48	0.0178 (15)	0.0306 (19)	0.0331 (19)	−0.0042 (14)	0.0074 (14)	−0.0001 (15)
C49	0.050 (3)	0.062 (3)	0.035 (2)	0.009 (2)	0.022 (2)	0.004 (2)

### Geometric parameters (Å, °)

**Table:**

Fe1—O5	2.001 (2)	C18—C19	1.439 (4)
Fe1—N3	2.038 (2)	C18—H18	0.9500
Fe1—N1	2.043 (2)	C19—C20	1.394 (4)
Fe1—N2	2.047 (2)	C20—C42	1.498 (4)
Fe1—N4	2.049 (2)	C21—C22	1.381 (4)
S1—O7	1.422 (3)	C21—C26	1.410 (4)
S1—O6	1.424 (3)	C22—C23	1.395 (4)
S1—O5	1.452 (2)	C22—H22	0.9500
S1—C49	1.828 (4)	C23—C24	1.395 (4)
F1—C49	1.324 (5)	C23—H23	0.9500
F2—C49	1.328 (6)	C24—C25	1.376 (5)
F3—C49	1.331 (5)	C25—C26	1.387 (4)
O1—C24	1.375 (4)	C25—H25	0.9500
O1—C27	1.423 (4)	C26—H26	0.9500
O2—C31	1.366 (3)	C27—H27A	0.9800
O2—C34	1.433 (4)	C27—H27B	0.9800
O3—C38	1.367 (4)	C27—H27C	0.9800
O3—C41	1.411 (4)	C28—C29	1.381 (4)
O4—C45	1.365 (3)	C28—C33	1.390 (4)
O4—C48	1.418 (4)	C29—C30	1.389 (4)
N1—C4	1.384 (4)	C29—H29	0.9500
N1—C1	1.386 (3)	C30—C31	1.383 (4)
N2—C6	1.383 (4)	C30—H30	0.9500
N2—C9	1.388 (4)	C31—C32	1.389 (4)
N3—C14	1.382 (4)	C32—C33	1.385 (4)
N3—C11	1.391 (4)	C32—H32	0.9500
N4—C19	1.377 (4)	C33—H33	0.9500
N4—C16	1.388 (4)	C34—H34A	0.9800
C1—C20	1.398 (4)	C34—H34B	0.9800
C1—C2	1.428 (4)	C34—H34C	0.9800
C2—C3	1.359 (4)	C35—C40	1.382 (5)
C2—H2	0.9500	C35—C36	1.389 (4)
C3—C4	1.434 (4)	C36—C37	1.380 (5)
C3—H3	0.9500	C36—H36	0.9500
C4—C5	1.398 (4)	C37—C38	1.390 (5)
C5—C6	1.405 (4)	C37—H37	0.9500
C5—C21	1.497 (4)	C38—C39	1.380 (5)
C6—C7	1.434 (4)	C39—C40	1.391 (5)
C7—C8	1.350 (4)	C39—H39	0.9500
C7—H7	0.9500	C40—H40	0.9500
C8—C9	1.431 (4)	C41—H41A	0.9800
C8—H8	0.9500	C41—H41B	0.9800
C9—C10	1.388 (4)	C41—H41C	0.9800
C10—C11	1.393 (4)	C42—C47	1.381 (4)
C10—C28	1.500 (4)	C42—C43	1.395 (4)
C11—C12	1.430 (4)	C43—C44	1.382 (4)
C12—C13	1.357 (4)	C43—H43	0.9500
C12—H12	0.9500	C44—C45	1.392 (5)
C13—C14	1.430 (4)	C44—H44	0.9500
C13—H13	0.9500	C45—C46	1.388 (5)
C14—C15	1.390 (4)	C46—C47	1.385 (4)
C15—C16	1.399 (4)	C46—H46	0.9500
C15—C35	1.493 (4)	C47—H47	0.9500
C16—C17	1.431 (4)	C48—H48A	0.9800
C17—C18	1.354 (4)	C48—H48B	0.9800
C17—H17	0.9500	C48—H48C	0.9800
			
O5—Fe1—N3	100.90 (10)	C23—C22—H22	119.0
O5—Fe1—N1	97.98 (9)	C22—C23—C24	119.0 (3)
N3—Fe1—N1	161.10 (10)	C22—C23—H23	120.5
O5—Fe1—N2	104.03 (10)	C24—C23—H23	120.5
N3—Fe1—N2	88.27 (10)	O1—C24—C25	116.0 (3)
N1—Fe1—N2	87.93 (9)	O1—C24—C23	123.8 (3)
O5—Fe1—N4	100.32 (10)	C25—C24—C23	120.2 (3)
N3—Fe1—N4	88.03 (10)	C24—C25—C26	120.2 (3)
N1—Fe1—N4	87.84 (9)	C24—C25—H25	119.9
N2—Fe1—N4	155.63 (10)	C26—C25—H25	119.9
O7—S1—O6	118.73 (17)	C25—C26—C21	120.8 (3)
O7—S1—O5	113.57 (15)	C25—C26—H26	119.6
O6—S1—O5	111.28 (15)	C21—C26—H26	119.6
O7—S1—C49	105.08 (19)	O1—C27—H27A	109.5
O6—S1—C49	104.7 (2)	O1—C27—H27B	109.5
O5—S1—C49	101.26 (18)	H27A—C27—H27B	109.5
C24—O1—C27	117.0 (2)	O1—C27—H27C	109.5
C31—O2—C34	115.8 (3)	H27A—C27—H27C	109.5
C38—O3—C41	116.9 (3)	H27B—C27—H27C	109.5
C45—O4—C48	116.9 (3)	C29—C28—C33	118.5 (3)
S1—O5—Fe1	137.11 (13)	C29—C28—C10	119.2 (3)
C4—N1—C1	105.7 (2)	C33—C28—C10	122.3 (3)
C4—N1—Fe1	126.53 (18)	C28—C29—C30	121.9 (3)
C1—N1—Fe1	127.30 (19)	C28—C29—H29	119.1
C6—N2—C9	106.1 (2)	C30—C29—H29	119.1
C6—N2—Fe1	126.95 (19)	C31—C30—C29	119.1 (3)
C9—N2—Fe1	125.77 (19)	C31—C30—H30	120.5
C14—N3—C11	105.4 (2)	C29—C30—H30	120.5
C14—N3—Fe1	125.80 (19)	O2—C31—C30	124.1 (3)
C11—N3—Fe1	125.9 (2)	O2—C31—C32	116.1 (3)
C19—N4—C16	105.7 (2)	C30—C31—C32	119.8 (3)
C19—N4—Fe1	127.4 (2)	C33—C32—C31	120.3 (3)
C16—N4—Fe1	125.57 (18)	C33—C32—H32	119.8
N1—C1—C20	126.1 (3)	C31—C32—H32	119.8
N1—C1—C2	110.1 (2)	C32—C33—C28	120.4 (3)
C20—C1—C2	123.8 (3)	C32—C33—H33	119.8
C3—C2—C1	107.1 (3)	C28—C33—H33	119.8
C3—C2—H2	126.5	O2—C34—H34A	109.5
C1—C2—H2	126.5	O2—C34—H34B	109.5
C2—C3—C4	107.4 (3)	H34A—C34—H34B	109.5
C2—C3—H3	126.3	O2—C34—H34C	109.5
C4—C3—H3	126.3	H34A—C34—H34C	109.5
N1—C4—C5	125.6 (3)	H34B—C34—H34C	109.5
N1—C4—C3	109.6 (2)	C40—C35—C36	118.2 (3)
C5—C4—C3	124.5 (3)	C40—C35—C15	120.6 (3)
C4—C5—C6	123.9 (3)	C36—C35—C15	121.2 (3)
C4—C5—C21	117.4 (3)	C37—C36—C35	121.0 (3)
C6—C5—C21	118.6 (3)	C37—C36—H36	119.5
N2—C6—C5	125.5 (3)	C35—C36—H36	119.5
N2—C6—C7	109.5 (2)	C36—C37—C38	120.4 (3)
C5—C6—C7	125.0 (3)	C36—C37—H37	119.8
C8—C7—C6	107.3 (3)	C38—C37—H37	119.8
C8—C7—H7	126.3	O3—C38—C39	124.7 (3)
C6—C7—H7	126.3	O3—C38—C37	116.2 (3)
C7—C8—C9	107.8 (3)	C39—C38—C37	119.1 (3)
C7—C8—H8	126.1	C38—C39—C40	120.0 (3)
C9—C8—H8	126.1	C38—C39—H39	120.0
C10—C9—N2	126.5 (3)	C40—C39—H39	120.0
C10—C9—C8	124.1 (3)	C35—C40—C39	121.3 (3)
N2—C9—C8	109.2 (3)	C35—C40—H40	119.3
C9—C10—C11	124.1 (3)	C39—C40—H40	119.3
C9—C10—C28	118.4 (3)	O3—C41—H41A	109.5
C11—C10—C28	117.3 (3)	O3—C41—H41B	109.5
N3—C11—C10	125.2 (3)	H41A—C41—H41B	109.5
N3—C11—C12	110.1 (3)	O3—C41—H41C	109.5
C10—C11—C12	124.7 (3)	H41A—C41—H41C	109.5
C13—C12—C11	107.0 (3)	H41B—C41—H41C	109.5
C13—C12—H12	126.5	C47—C42—C43	118.0 (3)
C11—C12—H12	126.5	C47—C42—C20	121.4 (3)
C12—C13—C14	107.5 (3)	C43—C42—C20	120.5 (3)
C12—C13—H13	126.2	C44—C43—C42	120.8 (3)
C14—C13—H13	126.2	C44—C43—H43	119.6
N3—C14—C15	125.6 (3)	C42—C43—H43	119.6
N3—C14—C13	110.0 (3)	C43—C44—C45	120.1 (3)
C15—C14—C13	124.4 (3)	C43—C44—H44	120.0
C14—C15—C16	124.0 (3)	C45—C44—H44	120.0
C14—C15—C35	117.7 (3)	O4—C45—C46	124.2 (3)
C16—C15—C35	118.2 (3)	O4—C45—C44	116.0 (3)
N4—C16—C15	125.9 (3)	C46—C45—C44	119.8 (3)
N4—C16—C17	110.0 (2)	C47—C46—C45	119.0 (3)
C15—C16—C17	124.1 (3)	C47—C46—H46	120.5
C18—C17—C16	107.1 (3)	C45—C46—H46	120.5
C18—C17—H17	126.4	C42—C47—C46	122.2 (3)
C16—C17—H17	126.4	C42—C47—H47	118.9
C17—C18—C19	107.3 (3)	C46—C47—H47	118.9
C17—C18—H18	126.3	O4—C48—H48A	109.5
C19—C18—H18	126.3	O4—C48—H48B	109.5
N4—C19—C20	126.2 (3)	H48A—C48—H48B	109.5
N4—C19—C18	109.8 (2)	O4—C48—H48C	109.5
C20—C19—C18	124.0 (3)	H48A—C48—H48C	109.5
C19—C20—C1	123.5 (3)	H48B—C48—H48C	109.5
C19—C20—C42	117.6 (3)	F1—C49—F2	109.3 (4)
C1—C20—C42	118.9 (3)	F1—C49—F3	107.7 (4)
C22—C21—C26	117.8 (3)	F2—C49—F3	108.5 (4)
C22—C21—C5	120.9 (3)	F1—C49—S1	109.3 (3)
C26—C21—C5	121.4 (3)	F2—C49—S1	111.9 (3)
C21—C22—C23	122.0 (3)	F3—C49—S1	110.1 (3)
C21—C22—H22	119.0		
			
O7—S1—O5—Fe1	−6.4 (3)	C19—N4—C16—C15	−180.0 (3)
O6—S1—O5—Fe1	130.7 (2)	Fe1—N4—C16—C15	−12.1 (4)
C49—S1—O5—Fe1	−118.5 (2)	C19—N4—C16—C17	−0.7 (3)
N3—Fe1—O5—S1	54.0 (2)	Fe1—N4—C16—C17	167.1 (2)
N1—Fe1—O5—S1	−126.8 (2)	C14—C15—C16—N4	−1.7 (5)
N2—Fe1—O5—S1	−36.9 (2)	C35—C15—C16—N4	−179.8 (3)
N4—Fe1—O5—S1	144.0 (2)	C14—C15—C16—C17	179.1 (3)
O5—Fe1—N1—C4	84.0 (2)	C35—C15—C16—C17	1.1 (5)
N3—Fe1—N1—C4	−98.4 (4)	N4—C16—C17—C18	1.1 (4)
N2—Fe1—N1—C4	−19.9 (2)	C15—C16—C17—C18	−179.6 (3)
N4—Fe1—N1—C4	−175.9 (2)	C16—C17—C18—C19	−1.0 (4)
O5—Fe1—N1—C1	−87.3 (2)	C16—N4—C19—C20	177.3 (3)
N3—Fe1—N1—C1	90.3 (4)	Fe1—N4—C19—C20	9.8 (4)
N2—Fe1—N1—C1	168.8 (2)	C16—N4—C19—C18	0.1 (3)
N4—Fe1—N1—C1	12.8 (2)	Fe1—N4—C19—C18	−167.5 (2)
O5—Fe1—N2—C6	−81.0 (3)	C17—C18—C19—N4	0.6 (4)
N3—Fe1—N2—C6	178.2 (3)	C17—C18—C19—C20	−176.7 (3)
N1—Fe1—N2—C6	16.7 (2)	N4—C19—C20—C1	−0.5 (5)
N4—Fe1—N2—C6	96.9 (3)	C18—C19—C20—C1	176.4 (3)
O5—Fe1—N2—C9	84.7 (3)	N4—C19—C20—C42	−179.5 (3)
N3—Fe1—N2—C9	−16.1 (3)	C18—C19—C20—C42	−2.6 (5)
N1—Fe1—N2—C9	−177.6 (3)	N1—C1—C20—C19	0.2 (5)
N4—Fe1—N2—C9	−97.5 (3)	C2—C1—C20—C19	178.3 (3)
O5—Fe1—N3—C14	76.4 (3)	N1—C1—C20—C42	179.2 (3)
N1—Fe1—N3—C14	−101.2 (4)	C2—C1—C20—C42	−2.7 (5)
N2—Fe1—N3—C14	−179.6 (3)	C4—C5—C21—C22	57.5 (4)
N4—Fe1—N3—C14	−23.7 (2)	C6—C5—C21—C22	−121.8 (3)
O5—Fe1—N3—C11	−81.4 (2)	C4—C5—C21—C26	−121.4 (3)
N1—Fe1—N3—C11	101.0 (3)	C6—C5—C21—C26	59.4 (4)
N2—Fe1—N3—C11	22.6 (2)	C26—C21—C22—C23	−1.1 (4)
N4—Fe1—N3—C11	178.5 (2)	C5—C21—C22—C23	180.0 (3)
O5—Fe1—N4—C19	84.7 (3)	C21—C22—C23—C24	0.4 (4)
N3—Fe1—N4—C19	−174.6 (3)	C27—O1—C24—C25	−171.8 (3)
N1—Fe1—N4—C19	−13.1 (3)	C27—O1—C24—C23	8.4 (4)
N2—Fe1—N4—C19	−93.2 (3)	C22—C23—C24—O1	−180.0 (3)
O5—Fe1—N4—C16	−80.6 (2)	C22—C23—C24—C25	0.2 (4)
N3—Fe1—N4—C16	20.2 (2)	O1—C24—C25—C26	−180.0 (3)
N1—Fe1—N4—C16	−178.3 (2)	C23—C24—C25—C26	−0.2 (4)
N2—Fe1—N4—C16	101.6 (3)	C24—C25—C26—C21	−0.6 (4)
C4—N1—C1—C20	178.0 (3)	C22—C21—C26—C25	1.2 (4)
Fe1—N1—C1—C20	−9.3 (4)	C5—C21—C26—C25	−179.9 (3)
C4—N1—C1—C2	−0.3 (3)	C9—C10—C28—C29	72.0 (4)
Fe1—N1—C1—C2	172.4 (2)	C11—C10—C28—C29	−103.4 (3)
N1—C1—C2—C3	−0.6 (4)	C9—C10—C28—C33	−111.3 (3)
C20—C1—C2—C3	−179.0 (3)	C11—C10—C28—C33	73.3 (4)
C1—C2—C3—C4	1.2 (3)	C33—C28—C29—C30	−0.2 (5)
C1—N1—C4—C5	−172.5 (3)	C10—C28—C29—C30	176.6 (3)
Fe1—N1—C4—C5	14.7 (4)	C28—C29—C30—C31	1.7 (5)
C1—N1—C4—C3	1.1 (3)	C34—O2—C31—C30	9.9 (4)
Fe1—N1—C4—C3	−171.7 (2)	C34—O2—C31—C32	−170.2 (3)
C2—C3—C4—N1	−1.5 (3)	C29—C30—C31—O2	177.9 (3)
C2—C3—C4—C5	172.2 (3)	C29—C30—C31—C32	−2.0 (5)
N1—C4—C5—C6	2.4 (5)	O2—C31—C32—C33	−179.1 (3)
C3—C4—C5—C6	−170.3 (3)	C30—C31—C32—C33	0.8 (5)
N1—C4—C5—C21	−176.8 (3)	C31—C32—C33—C28	0.7 (5)
C3—C4—C5—C21	10.5 (4)	C29—C28—C33—C32	−1.0 (4)
C9—N2—C6—C5	−175.7 (3)	C10—C28—C33—C32	−177.7 (3)
Fe1—N2—C6—C5	−7.8 (4)	C14—C15—C35—C40	−84.6 (4)
C9—N2—C6—C7	2.1 (3)	C16—C15—C35—C40	93.6 (4)
Fe1—N2—C6—C7	170.1 (2)	C14—C15—C35—C36	95.2 (4)
C4—C5—C6—N2	−6.0 (5)	C16—C15—C35—C36	−86.6 (4)
C21—C5—C6—N2	173.2 (3)	C40—C35—C36—C37	1.0 (5)
C4—C5—C6—C7	176.5 (3)	C15—C35—C36—C37	−178.8 (3)
C21—C5—C6—C7	−4.4 (5)	C35—C36—C37—C38	−0.1 (5)
N2—C6—C7—C8	−1.5 (4)	C41—O3—C38—C39	16.7 (5)
C5—C6—C7—C8	176.4 (3)	C41—O3—C38—C37	−164.1 (3)
C6—C7—C8—C9	0.2 (4)	C36—C37—C38—O3	−179.7 (3)
C6—N2—C9—C10	173.3 (3)	C36—C37—C38—C39	−0.4 (5)
Fe1—N2—C9—C10	5.2 (5)	O3—C38—C39—C40	179.2 (3)
C6—N2—C9—C8	−2.0 (3)	C37—C38—C39—C40	0.0 (5)
Fe1—N2—C9—C8	−170.1 (2)	C36—C35—C40—C39	−1.4 (5)
C7—C8—C9—C10	−174.3 (3)	C15—C35—C40—C39	178.4 (3)
C7—C8—C9—N2	1.1 (4)	C38—C39—C40—C35	1.0 (5)
N2—C9—C10—C11	7.3 (5)	C19—C20—C42—C47	−70.0 (4)
C8—C9—C10—C11	−178.1 (3)	C1—C20—C42—C47	110.9 (3)
N2—C9—C10—C28	−167.8 (3)	C19—C20—C42—C43	107.7 (3)
C8—C9—C10—C28	6.9 (5)	C1—C20—C42—C43	−71.4 (4)
C14—N3—C11—C10	179.4 (3)	C47—C42—C43—C44	3.0 (4)
Fe1—N3—C11—C10	−19.1 (4)	C20—C42—C43—C44	−174.8 (3)
C14—N3—C11—C12	−1.7 (3)	C42—C43—C44—C45	−0.1 (5)
Fe1—N3—C11—C12	159.8 (2)	C48—O4—C45—C46	4.5 (4)
C9—C10—C11—N3	0.0 (5)	C48—O4—C45—C44	−174.5 (3)
C28—C10—C11—N3	175.2 (3)	C43—C44—C45—O4	176.0 (3)
C9—C10—C11—C12	−178.7 (3)	C43—C44—C45—C46	−3.0 (5)
C28—C10—C11—C12	−3.6 (5)	O4—C45—C46—C47	−175.8 (3)
N3—C11—C12—C13	1.3 (4)	C44—C45—C46—C47	3.2 (5)
C10—C11—C12—C13	−179.8 (3)	C43—C42—C47—C46	−2.8 (5)
C11—C12—C13—C14	−0.4 (4)	C20—C42—C47—C46	174.9 (3)
C11—N3—C14—C15	−178.9 (3)	C45—C46—C47—C42	−0.2 (5)
Fe1—N3—C14—C15	19.6 (4)	O7—S1—C49—F1	59.7 (4)
C11—N3—C14—C13	1.4 (3)	O6—S1—C49—F1	−66.1 (4)
Fe1—N3—C14—C13	−160.1 (2)	O5—S1—C49—F1	178.1 (3)
C12—C13—C14—N3	−0.7 (4)	O7—S1—C49—F2	−61.5 (3)
C12—C13—C14—C15	179.7 (3)	O6—S1—C49—F2	172.7 (3)
N3—C14—C15—C16	−2.2 (5)	O5—S1—C49—F2	56.9 (3)
C13—C14—C15—C16	177.4 (3)	O7—S1—C49—F3	177.8 (3)
N3—C14—C15—C35	175.9 (3)	O6—S1—C49—F3	52.0 (4)
C13—C14—C15—C35	−4.5 (5)	O5—S1—C49—F3	−63.8 (4)
